# Voltage-induced magnetization dynamics in CoFeB/MgO/CoFeB magnetic tunnel junctions

**DOI:** 10.1038/srep42511

**Published:** 2017-02-17

**Authors:** Katsuya Miura, Shin Yabuuchi, Masaki Yamada, Masahiko Ichimura, Bivas Rana, Susumu Ogawa, Hiromasa Takahashi, Yasuhiro Fukuma, Yoshichika Otani

**Affiliations:** 1 Research and Development Group, Hitachi, Ltd., 1-280 Higashi-koigakubo, Kokubunji-shi, Tokyo 185-8601, Japan; 2 Center for Emergent Matter Science, RIKEN, 2-1 Hirosawa, Wako 351-0198, Japan; 3 Frontier Research Academy for Young Researchers, Kyushu Institute of Technology, 680-4 Kawazu, Iizuka 820-8502, Japan; 4 Institute for Solid State Physics, University of Tokyo, Kashiwa 277-858, Japan

## Abstract

Recent progress in magnetic tunnel junctions (MTJs) with a perpendicular easy axis consisting of CoFeB and MgO stacking structures has shown that magnetization dynamics are induced due to voltage-controlled magnetic anisotropy (VCMA), which will potentially lead to future low-power-consumption information technology. For manipulating magnetizations in MTJs by applying voltage, it is necessary to understand the coupled magnetization motion of two magnetic (recording and reference) layers. In this report, we focus on the magnetization motion of two magnetic layers in MTJs consisting of top layers with an in-plane easy axis and bottom layers with a perpendicular easy axis, both having perpendicular magnetic anisotropy. According to rectified voltage (*V*_rec_) measurements, the amplitude of the magnetization motion depends on the initial angles of the magnetizations with respect to the VCMA direction. Our numerical simulations involving the micromagnetic method based on the Landau-Lifshitz-Gilbert equation of motion indicate that the magnetization motion in both layers is induced by a combination of VCMA and transferred angular momentum, even though the magnetic easy axes of the two layers are different. Our study will lead to the development of voltage-controlled MTJs having perpendicular magnetic anisotropy by controlling the initial angle between magnetizations and VCMA directions.

Electron-spin-related phenomena have been attracting much attention because of their potential for enabling innovation in information processing technologies such as magnetic random access memory (MRAM)^1^,^2^,^3^ and nonvolatile logic chips. The essential technology to enable such innovation is manipulating magnetizations with low power consumption, which can contribute to sustainable green information technology world. Spin-transfer torque (STT)^4^,^5^ induced by spin-polarized current or magnetic (Oersted) fields^6^,^7^ induced by charge current are conventionally used to manipulate or switch magnetizations. However, power consumption cannot be reduced beyond a certain level for the current-controlled manipulation of magnetizations due to inherent Joule heating.

In addition to STT magnetization switching, the discovery of perpendicular magnetic anisotropy (PMA) at the interface between CoFeB and MgO^8^,^9^, which are widely used in magnetic tunnel junctions (MTJs)^8^,^9^,^10^,^11^,^12^ due to their giant tunnel magnetoresistance (TMR)^13^, is substantial progress in developing practical MRAM. To further reduce power consumption, many different trials for manipulating magnetic properties by voltage have been conducted, including voltage control of magnetostriction in multilayered stacks with piezoelectric materials^14^,^15^, Currie temperature in ferromagnetic semiconductors^16^,^17^, magnetoelectric effect in multiferroic materials^18^ or ferroelectric/ferromagnetic heterostructures^19^, and magnetic anisotropy^20^,^21^.

It has recently been reported that the ferromagnetic resonance (FMR) in MTJs with a perpendicular magnetic easy axis to the plane (p-MTJs) can be induced by applying radio frequency (RF) voltage due to voltage-controlled magnetic anisotropy (VCMA)^22^,^23^,^24^,^25^,^26^. Thus, the power consumption in MRAM can be significantly reduced if magnetizations in MTJs are fully controlled by applying voltage.

To develop voltage-controlled MTJs, the coupled motion of two magnetizations in MTJs must be considered because MTJs have two magnetic layers (recording and reference) and those two magnetizations are coupled to each other through interlayer dipolar coupling. To understand the magnetization motion in the two magnetic layers under applied voltage, we conduct rectified voltage (*V*_rec_) measurements^27^,^28^ in MTJs that have a relatively thicker top CoFeB layers with an in-plane magnetic easy axis and ultrathin bottom CoFeB layers with a perpendicular magnetic easy axis^8^, and conduct numerical micromagnetic simulations based on the Landau-Lifshitz-Gilbert (LLG) equation of motion by taking into account VCMA on a model sample consisting of two CoFeB layers.

## Results and Discussion

**Figure 1:**
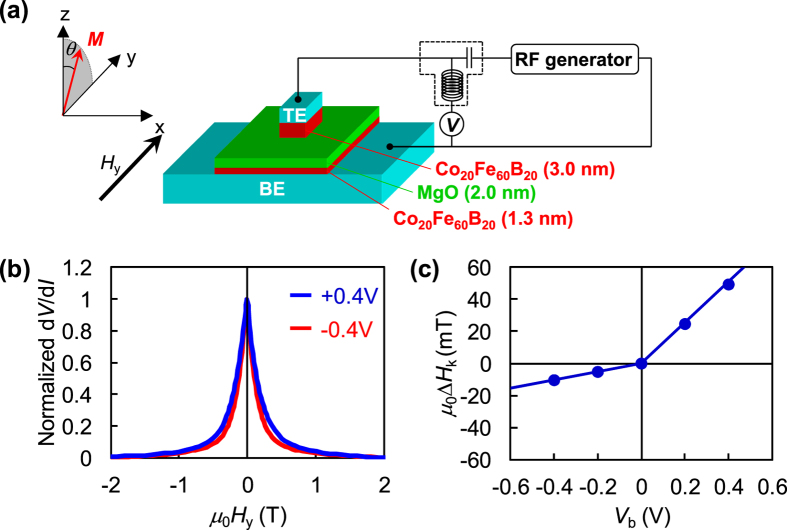
Schematic representation of sample structure and experimental results of VCMA magnitude. (**a**) Schematic illustration of CoFeB/MgO-based MTJ structure used in this study and experimental setup for rectified voltage measurements. (**b**) Normalized d*V*/d*I* as function of *H*_y_ under |*V*_b_| = 0.4 V. (**c**) Dependence of *μ*_0_Δ*H*_k_ on *V*_b_, from which we obtain *μ*_0_Δ*H*_k_/*V* of 127.1 (26.0) mT/V in positive (negative) *V*_b_ region.

We prepare MTJs having 3-nm-thick top CoFeB layers (reference layer) with the in-plane magnetic easy axis and 1.3-nm-thick bottom CoFeB layers (free layer) with the perpendicular magnetic easy axis^8^ (see Methods), as schematically shown in Fig. 1(a), along with the coordinate system. The CoFeB layers are separated by a 2.0-nm-thick MgO layer.

The reason for having PMA in the thin CoFeB layers has been attributed to the hybridization of Fe 3*d* and O 2*p* orbitals from the first-principles calculation^29^. The basic idea of VCMA is the modulation of the charge or spin density in the 3*d* orbitals by voltage or electric field^30^,^31^,^32^. Relative changes in spin density in the occupied orbitals can cause a change in PMA.

Both (top and bottom) ferromagnetic layers in our MTJs have interfacial PMA because they have an interface with the MgO layer. However, the direction of the easy axis strongly depends on the thickness of the ferromagnetic layer (inversely proportional to thickness)^33^. For the bottom layer, the PMA field overcomes the demagnetizing field to have net effective PMA. Therefore, the bottom layer has a perpendicular magnetic easy axis. However, the PMA value in the top layer is less than the demagnetizing field. Therefore, the top layer has an in-plane magnetic easy axis^33^.

To evaluate the VCMA field, we measure the differential resistance (d*V*/d*I*) of the MTJ as a function of an external magnetic field along the *y*-axis (*H*_y_) under various bias voltages (*V*_b_) ranging from −0.4 to +0.4 V. A positive voltage means the bottom electrode has positive potential with respect to the top electrode of the MTJ.

The curves of normalized d*V*/d*I* at *V*_b_ = −0.4 V (red line) and +0.4 V (blue line) are plotted as functions of *H*_y_ in Fig. 1(b). At *μ*_0_*H*_y_ = −2.0 T, the two curves show their minimum values after saturation, indicating that the magnetizations in both (top and bottom) CoFeB layers are along the negative *y*-axis. As *H*_y_ increases to zero, the curves of d*V*/d*I* under |*V*_b_| = 0.4 V increase and reach maximum values at the zero magnetic field, indicating that the magnetization in the bottom CoFeB layer is almost aligned along the out-of-plane direction. When *H*_y_ turns to a positive value, the magnetization in the top CoFeB layer starts to align towards the positive *y*-axis. Then, the curves of d*V*/d*I* decrease with the increase in *H*_y_ for both bias voltages and reach minimum values again, indicating that the magnetizations of both CoFeB layers again lie along the in-plane direction (+*y*-axis).

The difference between the two curves in Fig. 1(b) is clear. The difference comes from the modulation of an interfacial PMA field (*H*_k_) by *V*_b_ through the VCMA effect. From the difference in *H*_k_ due to the applied *V*_b_, the magnitude of VCMA, *i.e*., *μ*_0_Δ*H*_k_/*V*, can be determined. We evaluate Δ*H*_k_, which are defined by the difference between *H*_k_(*V*_b_) and *H*_k_(0) (by using the method mentioned in pervious studies 24, 34), at |*V*_b_| = 0, 0.2, and 0.4 V, and Fig. 1(c) plots Δ*H*_k_ as a function of *V*_b_. The gradients of *μ*_0_Δ*H*_k_ are different for positive and negative *V*_b_. At positive (negative) *V*_b_, *μ*_0_Δ*H*_k_/*V*_b_ is 127.1 (26.0) mT/V. The possible reason behind the difference between gradients under positive and negative *V*_b_ has been discussed^29^,^34^. According to these reports, the different thicknesses of the two CoFeB layers in the MTJs may show asymmetric dependence of PMA on positive and negative *V*_b_.

**Figure 2:**
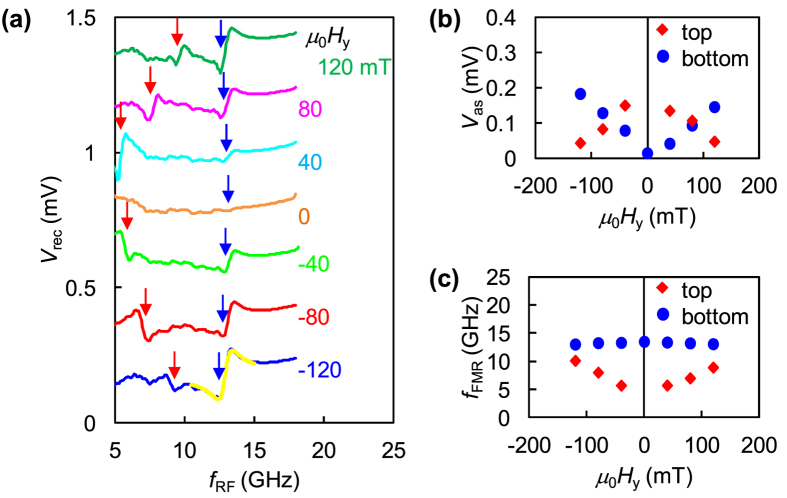
Experimental results of rectified voltage. (**a**) *V*_rec_ as function of *f*_RF_ with *V*_RF_ = 0.4 V at *μ*_0_|*H*_y_| of 0, 40, 80, and 120 mT. *V*_rec_ curves at different *H*_y_ are vertically offset by 0.25 mV. FMR peaks indicated by red arrows come from top CoFeB layer, and peaks indicated by blue arrows come from bottom CoFeB layer. Yellow line represents fitting curve obtained using Eq. (1). (**b**) Dependence of *V*_as_ on *H*_y_. Red (blue) symbols represent *V*_as_ of top (bottom) layer. (**c**) Dependence of *f*_FMR_ on *H*_y_. Red (blue) symbols represent *V*_as_ of top (bottom) layer.

Figure 2(a) shows the dependencies of *V*_rec_ on the excitation frequency (*f*_RF_) at an amplitude of RF voltage *V*_RF_ = 0.4 V. A static magnetic field is applied along the *y*-axis ranging from −120 to +120 mT (see Methods). In this figure, each curve of *V*_rec_ artificially shifts by 0.25 mV vertically.

The curves of *V*_rec_ show two FMR peaks^35^. One of the FMR peaks shows a strong dependence of resonance frequency (*f*_FMR_) on *H*_y_ (indicated by red arrows), whereas the other peak shows a slight change in *f*_FMR_ with *H*_y_ (indicated by blue arrows). The former peak corresponds to the FMR of the top CoFeB layer, whereas the latter peak corresponds to that of the bottom CoFeB layer.

**Figure 3:**
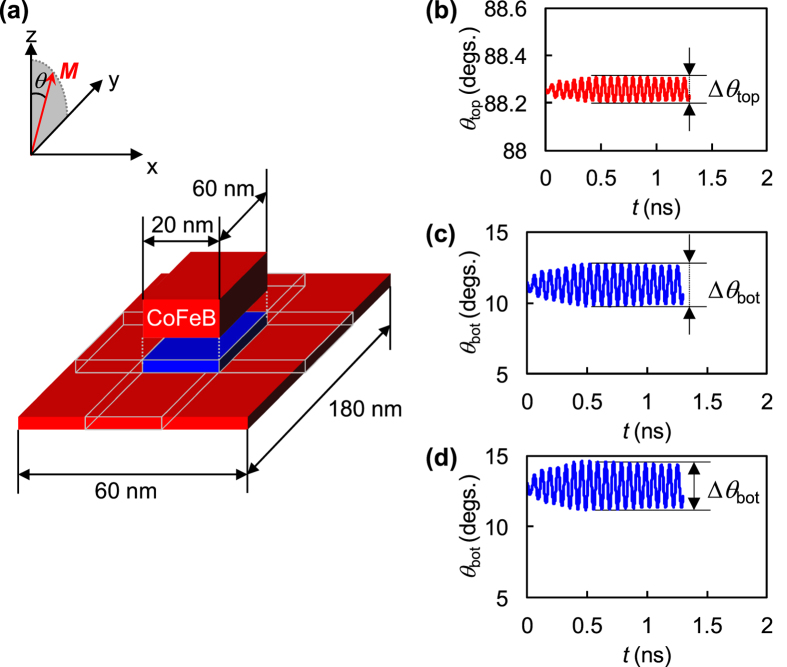
Schematic of model sample for micromagnetic simulations and simulation results. (**a**) Schematic illustration of model samples with top and bottom CoFeB layers. Blue indicates area where RF voltage is applied to bottom CoFeB layer. Coordinate system is also shown. (**b**,**c**) *θ*_top_ and *θ*_bot_ as functions of *t* under applied VCMA field of 30 mT and *f*_RF_ = 15 GHz for sample that includes both top and bottom CoFeB layers. (**d**) *θ*_bot_ as function of *t* under applied VCMA field of 30 mT and *f*_RF_ = 15 GHz for sample without top CoFeB layer. In simulations, offset field *H*_offset_ to *H*_eff_ is added for correcting zero point of VCMA on *V*_b_.

The difference between the dependencies of *f*_FMR_ on *H*_y_ in the two layers can be explained as follows. The *f*_FMR_ of a ferromagnetic layer depends on the effective magnetic field of the layer. The effective field of the top CoFeB layer monotonically increases with |*H*_y_| due to the in-plane easy-axis of magnetization. On the other hand, the effective field of the bottom CoFeB layer does not vary significantly in the range of applied magnetic field due to the presence of strong PMA. In the case at *μ*_0_|*H*_y_| = 120 mT, the magnetization of the bottom CoFeB layer tilts only up to 13° from the *z*-axis (Fig. 3(c)).

The line shapes of resonance peaks (Fig. 2(a)) in the bottom and top layers can be fitted by the sum of the symmetric and anti-symmetric Lorentzians given by^24^,^35^,^36^:


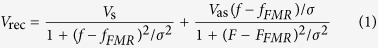


where *V*_s_ (*V*_as_) is the symmetric (anti-symmetric) term of signal amplitude, and *σ* is the half width at half maximum. Here, *V*_s_ originates from the STT-induced FMR, whereas *V*_as_ comes from the FMR induced by modulation of the effective field through VCMA and field-like torque (FLT)^24^,^36^,^37^.

Equation (1) fits the experimental resonance peaks quite well. Figure 2(a) shows an example of such fitting for *V*_rec_ at *μ*_0_*H*_y_ = −120 mT for the bottom CoFeB layer (yellow line). According to the results of fitting, *V*_as_ is around ten times larger than *V*_s_ for both layers. Therefore, the contribution of VCMA torque is dominant over the STT because the contribution of the STT is too small to induce FMR due to the large resistance of the 2.0-nm-thick MgO barrier (current density is less than 1.0 × 10^6^ A/m^2^). Similarly, the FLT is also expected to be much smaller than VCMA torque^28^.

Figure 2(b) shows the *V*_as_ of the top and bottom layers as functions of *H*_y_. As shown in Fig. 2(b), the *V*_as_ of the top layer is maximum at *H*_y_ = 0 and decreases with |*H*_y_|. This is because the equilibrium direction of the magnetization slightly tilts from the *x*-y plane at *H*_y_ = 0 due to the interlayer dipolar coupling and aligns toward the *y*-direction as *H*_y_ increases (see Fig. 3(b)). The small angle tilted from the *x*-*y* plane contributes to the magnetization motion in this case. Therefore, *V*_as_ strongly depends on the initial configuration of the magnetizations, *i.e*., having a finite angle between the magnetization direction and *z*-axis at *H*_y_ = 0. In contrast, the *V*_as_ of the bottom layer is almost zero at *H*_y_ = 0 and increases monotonically with |*H*_y_|. The reason for the *V*_as_ behaviour of the top and bottom layers is discussed in detail with the help of micromagnetic simulations in the last section of this report.

Figure 2(c) plots the *f*_FMR_ of the top and bottom layers obtained from the fitting as functions of *H*_y_. As shown in Fig. 2(c), the *f*_FMR_ of the top layer increases with |*H*_y_|, reflecting the increase in the effective field, as mentioned above. The *f*_FMR_ of the bottom layer slightly decreases as |*H*_y_| increases because of the weak dependence of the effective field on *H*_y_ in the out-of-plane magnetization configuration.

From the experiments, we find that the dependence of the magnitude of the magnetization motion on *H*_y_ is different between the top and bottom CoFeB layers. The results indicate that the amplitude of the magnetization motion in MTJs can be potentially controlled by the initial configuration of two magnetizations in the top and bottom magnetic layers.

The *V*_rec_ can be expressed as a time-averaged value of the product of oscillating magneto-resistance due to the precession of magnetization via the VCMA and RF tunnel current as^24^,^36^


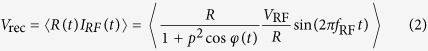


where *R* is the static resistance of an MTJ, *p* is spin polarization, and *φ(t*) is the time-dependent relative angle between the magnetizations in the top and bottom CoFeB layers. The term *R(t) = R*/(1 + *p*^2^cos*φ(t*)) represents the time-dependent resistance due to oscillation of *φ*, and *I*_RF_(*t*) = *V*_RF_sin(2*πf*_RF_*t*)/*R* represents the time-dependent tunnel current. As shown in Eq. (2), *V*_rec_ is represented by the time variation of *φ* when *V*_RF_ is applied. To obtain the magnetization motion of the top and bottom CoFeB layers through the time variation of *φ*, therefore, we conduct micromagnetic simulations based on the LLG equation of motion by taking into account the VCMA effect.

The LLG equation of motion is given by


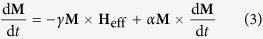


where the STT term can be ignored because of its small contribution in the MTJ. In this equation, **M** is magnetization, *γ *= 1.7 × 10^−11^ is the gyromagnetic ratio, *α *= 0.03 is the Gilbert damping constant^33^, and **H**_**eff**_ is the effective magnetic field. The effective field in the perpendicular direction (*H*_eff_)_z_ = [*H*_k_ + Δ*H*_k_sin(2*πf*_RF_*t*)]cos*θ* includes contributions from *H*_k_ and the time-dependent modulation of the PMA field Δ*H*_k_sin(2*πf*_RF_*t*) by applying *V*_RF_. Equation (3) is numerically solved using the object-oriented micromagnetic framework (OOMMF) simulator^38^.

The model samples for the simulations have top (with the in-plane magnetic easy axis) and bottom (with the perpendicular magnetic easy-axis) CoFeB layers separated by a distance of 2.0 nm (Fig. 3(a)) to mimic the experimentally measured MTJ with a 2.0-nm-thick insulating MgO layer (see Methods). The *μ*_0_Δ*H*_k_ is taken as 30 mT, which corresponds to the experimentally applied *V*_RF_ of 0.4 V. In all our simulations, a static magnetic field of 120 mT is applied along the *y*-axis.

The simulation results of the polar angles (angles with respect to the *z*-axis) of the magnetizations in the top and bottom CoFeB layers, *θ*_top_ and *θ*_bot_, are plotted as functions of an elapsed time *t* when applying RF voltage with *f*_RF_ = 15.0 GHz in Fig. 3(b) and (c), respectively. In this case, the VCMA field is applied only to the area of the bottom CoFeB layer just below the top CoFeB layer (blue area in Fig. 3(a)). The *θ*_top_ is obtained from the averaged magnetization over the top CoFeB layer and *θ*_bot_ from the averaged magnetization over the blue area in the bottom CoFeB layer.

As shown in Fig. 3(b), at *t* = 0, the magnetization in the top CoFeB layer makes a small angle (~1.8°) with respect to the *x*-*y* plane due to the interlayer dipolar coupling with the bottom CoFeB layer. The magnetization of the bottom CoFeB layer is also tilted from the perpendicular direction by around 11° (Fig. 3(c)), reflecting the effect of the sum of *H*_y_ and interlayer dipolar coupling.

As *t* increases, the magnetization in the bottom CoFeB layer oscillates at a frequency of 15 GHz due to VCMA. The magnetization in the top CoFeB layer also oscillates despite the fact that VCMA is not taken into account in the simulation model and 15 GHz is not the resonance frequency of the top layer. These results indicate that a small amount of angular momentum of the bottom CoFeB layer is transferred to the top CoFeB layer due to the dynamic dipolar coupling between them while they are separated by a distance of 2.0 nm^39^.

As shown in Fig. 3(b) and (c), Δ*θ*_top_ and Δ*θ*_bot_ are defined as amplitudes of oscillation angles of magnetizations in the top and bottom CoFeB layers, respectively. At *f*_RF_ = 15.0 GHz, Δ*θ*_bot_ = 2.9° and Δ*θ*_top_ = 0.1° are obtained. To confirm the magnitude of the transferred angular momentum, we also conduct a simulation by using a model sample with a bottom CoFeB layer only. Figure 3(d) plots *θ*_bot_ as a function of *t*. In this case, we obtain Δ*θ*_bot_ = 3.4°, which is larger than that in the sample with the top CoFeB layer. This result indicates that in our simulation model, the angular momentum is transferred from bottom to top CoFeB layers by more than 3% in angle equivalent. Similarly, the angular momentum induced in the excitation area (blue area in Fig. 3(a)) may also be transferred to the remaining area in the bottom CoFeB layer (red area in the bottom CoFeB layer in Fig. 3(a)).

**Figure 4:**
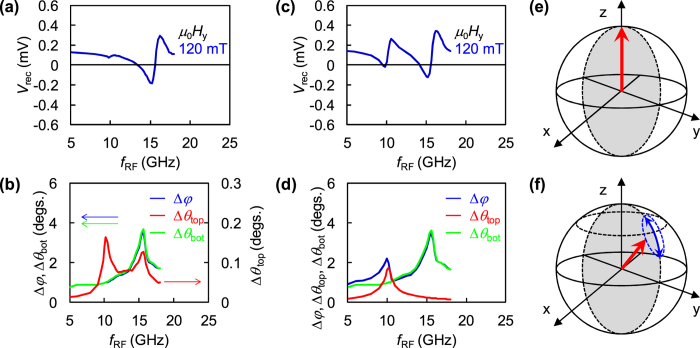
Micromagnetic simulation results and schematics of magnetization dynamics under VCMA. (**a**) Simulation results of *V*_rec_ as function of *f*_RF_ when VCMA is applied to bottom CoFeB layer. (**b**) Δ*φ* (blue line), Δ*θ*_top_ (red line), and Δ*θ*_bot_ (green line) as functions of *f*_RF_ when VCMA is applied to bottom CoFeB layer. (**c**) Simulation results of *V*_rec_ as function of *f*_RF_ when VCMA is applied to both top and bottom CoFeB layers. (**d**) Δ*φ* (blue line), Δ*θ*_top_ (red line), and Δ*θ*_bot_ (green line) as functions of *f*_RF_ when VCMA is applied to both top and bottom CoFeB layers. (**e**,**f**) Schematic figures of magnetization dynamics in bottom CoFeB layer without and with *H*_y_, respectively.

We conduct the simulations at various *f*_RF_ at *μ*_0_*H*_y_ = 120 mT using a model sample with the top and bottom CoFeB layers and plotted *V*_rec_ calculated from simulation results as a function of *f*_RF_ (Fig. 4(a)). Note that the VCMA field is applied only to the bottom CoFeB layer in this simulation.

The simulated *V*_rec_ reproduces the experimentally obtained FMR signal at 15.6 GHz from the bottom CoFeB layer, as shown in Fig. 2(a). However, the simulated resonance frequencies of the top and bottom CoFeB layers are larger than the experimental values due to the smaller dimensions of the model sample for simulation compared to the dimensions of the experimental sample^40^. The height of the resonance peak at around 10 GHz, which comes from the oscillation in the top CoFeB layer, is much smaller than that at 15.6 GHz, unlike the experimental results.

The possible reason for the small amplitude of the resonance peak at around 10 GHz is explained as follows. The simulated Δ*φ* (blue line), which is the oscillation amplitude of the relative angle between magnetizations of the top and bottom layers, is plotted as a function of *f*_RF_ together with Δ*θ*_top_ (red line) and Δ*θ*_bot_ (green line) in Fig. 4(b). Both Δ*φ* and Δ*θ*_bot_ increase as *f*_RF_ increases and reach a maximum of around 4° when *f*_RF_ approaches *f*_FMR_ of 15.6 GHz. In the case of the top CoFeB layer, Δ*θ*_top_ shows a maximum value of 0.2° at 10.2 GHz. This peak corresponds to the *f*_FMR_ of the top CoFeB layer, as shown in Fig. 2(a), which is generated by the transfer of angular momentum from the bottom CoFeB layer, although the Δ*θ*_top_ is not large enough to reproduce the experimentally obtained peak.

The Δ*θ*_top_ shows another peak (0.1°) at 15.6 GHz, which corresponds to the *f*_FMR_ of the bottom CoFeB layer. This peak is considered to be induced by the FMR mode of the bottom CoFeB layer. Therefore, we conclude that the top CoFeB layer with the in-plane magnetic easy axis can be excited by the transfer of angular momentum from the bottom CoFeB layer. However, the signal is much smaller than that of the experiment.

We also conduct a simulation by taking into account the VCMA in the top CoFeB layer. It is also possible to excite the top layer by VCMA, like the bottom layer^25^, since the top CoFeB layer has an interfacial PMA of about 0.8 T (<*M*_s_), and the magnetization of the top CoFeB layer makes a small angle with in-plane direction under the equilibrium (static) condition (Fig. 3(b)). To reproduce the *V*_rec_ signal from the top CoFeB layer at around 10.0 GHz, the VCMA in the top CoFeB layer is included in the simulations in addition to that in the bottom CoFeB layer.

The magnetization of the top CoFeB layer makes a small angle with the *x*-*y* plane. Because the VCMA field decreases in proportional to cos*θ*^25^,^41^, the VCMA field applied to the top CoFeB layer must be smaller than that in the bottom CoFeB layer. In this model, the magnitude of the VCMA field applied to the top CoFeB layer is selected to be Δ*H*_k_ = −1.5 mT, which is comparable to the estimated VCMA field considering factor cos*θ*. The Δ*H*_k_ in the z-direction is applied to the lower 1.0-nm region of the top CoFeB layer since VCMA is effective only at the interface area.

The simulation results of *V*_rec_ are plotted as a function of *f*_RF_ at *μ*_0_*H*_y_ = 120 mT in Fig. 4(c). The simulated *V*_rec_ agrees well with the line shape of the experimentally obtained *V*_rec_ plotted in Fig. 2(a), showing two FMR peaks. Figure 4(d) illustrates the dependencies of Δ*φ* (blue line), Δ*θ*_top_ (red line), and Δ*θ*_bot_ (green line) on *f*_RF_. In this figure, Δ*φ* shows two peaks, at 10.2 and 15.6 GHz, reflecting the FMR signals from the top and bottom CoFeB layers. In this simulation, therefore, the top layer is predominantly excited by VCMA, like the bottom layer.

Finally, we discuss the magnetization motion of the top and bottom CoFeB layers generated by RF voltage in detail. The *V*_as_ in the top CoFeB layer is maximum at *H*_y_ = 0 and decreases as |*H*_y_| increases. In the case of the top CoFeB layer, it is important to have a small angle between the magnetization direction and *x-y* plane for the magnetization motion, as mentioned above.

The *V*_as_ in the bottom CoFeB layer increases as |*H*_y_| increases in the magnetic-field range from −120 to +120 mT, while the *V*_as_ is almost zero at *H*_y_ = 0, as shown in Fig. 2(c). The dependence of *V*_as_ on *H*_y_ is consistent with the results presented in a previous study^25^. The possible reason for this behavior of *V*_as_ is explained as follows.

In the absence of *H*_y_, the equilibrium direction of magnetization of the bottom CoFeB layer is in the *z*-axis. In this situation, the magnetization does not show apparent motion because VCMA corresponds to a modulation along the *z*-direction. As a result, the magnetization (red arrow) remains in the *z*-direction even under applied RF voltage, as shown in Fig. 4(e). In contrast, the equilibrium direction of the magnetization tilts toward the *y*-direction if *H*_y_ is applied. When RF voltage is applied, the VCMA field modulates the magnetization toward the *z*-direction. Therefore, the magnetization in this situation shows reciprocal motion in the *y*-*z* plane in addition to precessional motion, as shown in Fig. 4(f). We have also confirmed that the precessional amplitude (cone angle of precession) increases with the increase in |*H*_y_| in the range of our measurement (not shown), which is consistent with our experimental results.

## Conclusion

We have investigated the magnetization motion in the two magnetic layers in MTJs under applied RF voltage by using both experimental measurements and micromagnetic simulations by taking into account VCMA. From the experiments, the dependence of the magnitude of the magnetization motion on *H*_y_ is different between the top and bottom CoFeB layers, and magnetization dynamics strongly depends on the initial angles of magnetizations with respect to the VCMA direction. From the simulation results, the magnetization motion in both the top and bottom CoFeB layers is induced by a combination of VCMA and transferred angular momentum, although the magnetization direction of the two layers is different. Our results will have a large impact on understanding the mechanism of magnetization dynamics in MTJs excited by VCMA and developing voltage-controlled MTJs having PMA by controlling the initial angle between magnetizations and VCMA directions.

## Methods

### Sample fabrication

The film-stacking structure used in this study is prepared on a thermally oxidized Si(001) substrate by RF sputtering at room temperature at a base pressure of 10^−9^ Torr. The structure consists of the following layers (from the substrate side; nominal thicknesses in nanometres are stated in parentheses): Ta (5)/Ru (10)/Ta (5)/Co_20_Fe_60_B_20_ (1.3)/MgO (2.0)/Co_20_Fe_60_B_20_ (3.0)/Ta (5)/Ru (5). Rectangular shaped MTJs, as shown in Fig. 1(a), are fabricated by a combination of photolithography, Ar-ion milling, and RF sputtering in a multistep fabrication method. First, the top CoFeB layer of 2 × 6 μm^2^ is prepared by photolithography followed by Ar^+^ ion milling down to the top MgO layer and subsequent deposition of Al_2_O_3_. In the second step, the bottom CoFeB layer with larger dimensions 40 × 40 μm^2^ is defined, keeping the former rectangular structure in the middle followed by Ar^+^ ion milling down to the Si substrate. Third, 80-nm-thick insulator Al_2_O_3_ was deposited by sputtering everywhere except at the extremities of the top and bottom CoFeB layers. Finally, contacts made of Au (100 nm) are prepared by electron beam evaporation. The fabricated MTJs are annealed at 300 °C in vacuum under a perpendicular magnetic field of 600 mT for one hour.

### Experimental measurement

The magnetization dynamics in CoFeB layers are excited by sending RF voltage through the capacitor port of a bias tee. The RF voltage (*V*_RF_) produces an RF electric field (*E*_RF_) at the interfaces of the top and bottom CoFeB/MgO layers. The *E*_RF_ modulates the interfacial PMA of both CoFeB layers. When the frequency of *V*_RF_ matches the *f*_FMR_ of any layer, the magnetization dynamics of that layer is excited. The magnetization dynamics produces an oscillatory TMR (depending upon the relative angle between the magnetizations of two layers) at the same frequency as that of the *V*_RF_. The mixing of an oscillatory TMR and small RF tunnel current generates finite DC voltage (rectified voltage), which is measured using a voltmeter connected to the DC port of the bias tee.

### Micromagnetic simulations

Two types of model samples are considered for the simulations. One has top and bottom CoFeB layers with thicknesses of 3.0 and 1.0 nm, respectively, as shown in Fig. 3(a). The mesh size is set to 2 × 2 × 1 nm^3^. The two CoFeB layers are arranged 2.0 nm apart. The MTJs are downscaled to reduce the computation time. The lateral dimensions of the bottom CoFeB layer is 60 × 180 nm^2^, and those of the MgO and top CoFeB layers is 20 × 60 nm^2^. For the other model sample, we chose only a single CoFeB layer with a thickness of 1.0 nm and area 60 × 180 nm^2^. We adopt the anisotropy energy density of the bottom CoFeB layer in the perpendicular direction of 1.2 MJ/m^3^, and the saturation magnetization *M*_s_ of 1.5 T, resulting in *μ*_0_*H*_k_ of around 500 mT. The ground state of magnetization is first prepared by applying a bias magnetic field along the *y*-axis. The dynamics is then excited by applying a sinusoidal RF magnetic field (Δ*H*_k_) equivalent to a PMA field modulated by applied *V*_RF_ of 0.4 V. For the bottom layer, Δ*H*_k_ is selected as 30 mT (calculated from the experimental results shown in Fig. 1(c)). As the PMA is only modulated in the area of the bottom CoFeB layer underneath the top layer (blue area in Fig. 3(a)), Δ*H*_k_ is applied only to the blue area of the bottom CoFeB layer. In some simulations, the dynamics of the top CoFeB layer are also exited by a much smaller value of Δ*H*_k_ = −1.5 mT along the *z*-axis. The reason behind this small value is explained in the main text.

## Additional Information

**How to cite this article:** Miura, K. *et al*. Voltage induced magnetization dynamics in CoFeB/MgO/CoFeB magnetic tunnel junctions. *Sci. Rep.* **7**, 42511; doi: 10.1038/srep42511 (2017).

**Publisher's note:** Springer Nature remains neutral with regard to jurisdictional claims in published maps and institutional affiliations.
